# Copper Film Modified Glassy Carbon Electrode and Copper Film with Carbon Nanotubes Modified Screen-Printed Electrode for the Cd(II) Determination

**DOI:** 10.3390/ma14185148

**Published:** 2021-09-08

**Authors:** Joanna Wasąg, Malgorzata Grabarczyk

**Affiliations:** 1 Department of Materials Engineering, Institute of Engineering and Technical Sciences, Faculty of Natural Sciences and Health, The John Paul II Catholic University of Lublin, 20-950 Lublin, Poland; 2 Department of Analytical Chemistry, Institute of Chemical Sciences, Faculty of Chemistry, Maria Curie-Sklodowska University, 20-031 Lublin, Poland; mgrabarc@poczta.umcs.lublin.pl

**Keywords:** copper modified electrode, carbon-based electrode materials, screen-printed electrode, electrochemical detection, stripping voltammetry, cadmium determination

## Abstract

A copper film modified glassy carbon electrode (CuF/GCE) and a novel copper film with carbon nanotubes modified screen-printed electrode (CuF/CN/SPE) for anodic stripping voltammetric measurement of ultratrace levels of Cd(II) are presented. During the development of the research procedure, several main parameters were investigated and optimized. The optimal electroanalytical performance of the working electrodes was achieved in electrolyte 0.1 M HCl and 2 × 10^−4^ M Cu(II). The copper film modified glassy carbon electrode exhibited operation in the presence of dissolved oxygen with a calculated limit of detection of 1.7 × 10^−10^ M and 210 s accumulation time, repeatability with RSD of 4.2% (*n* = 5). In the case of copper film with carbon nanotubes modified screen-printed electrode limit of detection amounted 1.3 × 10^−10^ M for accumulation time of 210 s and with RSD of 4.5% (*n* = 5). The calibration curve has a linear range in the tested concentration of 5 × 10^−10^–5 × 10^−7^ M (r = 0.999) for CuF/GCE and 3 × 10^−10^–3 × 10^−7^ M (r = 0.999) for CuF/CN/SPE with 210 s accumulation time in both cases. The used electrodes enable trace determination of cadmium in different environmental water samples containing organic matrix. The validation of the proposed procedures was carried out through analysis certified reference materials: TM-25.5, SPS-SW1, and SPS-WW1.

## 1. Introduction

This work developed a novel voltammetric procedure for determination of cadmium using two types of working electrode: a copper film with carbon nanotubes modified screen-printed electrode (CuF/CN/SPE) and a copper film modified glassy carbon electrode (CuF/GCE). For the first time, copper modified electrodes were used to determine ultratrace amounts of Cd(II) ions. The use of copper as a film on the surface of the working electrode is a very important aspect, as now a lot of emphasis is placed on the development of a new type of film electrodes using non-toxic metals. Copper is non-toxic and allowed us to obtain very low detection limit for cadmium. Importantly, this work was also created to draw the attention of scientists to this type of copper electrodes, which has been practically unused until now, but is proving to be a powerful tool in the trace analysis of many metal ions. At work, it is also important to determine cadmium on two different working electrodes under almost identical measurement conditions, which allows the measurements to be transferred to field conditions and the results obtained with both methods can be compared. The procedure was first developed and optimized for the CuF/GCE working electrode and then successful measurements were carried out using the obtained parameters with the novel modified CuF/CN/SPE electrode. As already mentioned, in the literature, we do not find too many works relating to the use of the copper film electrode [1,2,3,4]. Copper film modified electrodes seem to be an excellent proposal for the determination of trace amounts of metals also in real samples. Such an electrode allows low detection limits to be obtained, and the fact that it is created in situ from the test solution significantly shortens the measurement time. The SPE electrode, on the other hand, has several advantages over conventional electrodes, such as the simplicity of use, commercial availability, low price, and the possibility of using it in field research as a portable sensor. The reproducibility and sensitiveness of these electrodes are very good, so they can replace classical solid electrodes in the analysis [5,6]. Their effectiveness in analysis gives a chance for their widespread and more frequent use [7,8,9,10]. These electrodes are also easily accessible to everyone. There is a wide variety of screen-printed electrode materials in the commercial industry, depending on the specific needs. They can be easily purchased and used in direct field analyzes.

Film metal modified electrodes have become more and more popular in recent years, especially as a replacement for toxic mercury electrodes. These electrodes can be generated on various substrates, but the most common is glassy carbon [1,2,4,11,12,13,14]. In recent years, more attention has been paid to screen-printed electrodes, which can be used either as direct working electrodes or as an attractive substrate for the generation of film metal electrodes. The sensitivity of screen-printed electrodes can be increased by the incorporation of desirable functional parameters or specific nanoparticles in the ink before the printing process. The screen-printed electrode used in this work is modified with carbon nanotubes (CN), which have a large surface, excellent electrical conductivity, and good chemical stability [15]. Carbon nanotubes exhibit better electrochemical performance than other carbon-based electrodes. In the literature, there are examples of the use of electrodes that are modified by carbon nanotubes. To name a few uses, they have been used for electrochemical oxidation of inorganic and organic compounds, including pharmaceuticals [16], and catalytic oxidation of thiols [11]. Various modifications with the use of copper are also known in the literature [17,18,19]. In our work, a copper coating is applied to the surface of the working electrode, which forms an integral part of the electrode. In other works, an interesting solution is the use of hybrid materials based on copper oxide successfully synthesized by an ultrasound sonochemical method and applied as an electrode material for supercapacitor applications [17]. Another interesting example can be the use of metal organic framework (MOF) derived Co-Al layered double hydroxide by Cr(VI) and Pb(II) ion adsorption [18]. These works give us an insight into the effectiveness of the practical use of copper-based materials as a diverse medium for the determination of many metal ions.

The aim of our research was to use modified copper film electrodes generated on various substrates, such as GCE and CN/SPE, and to develop competitive procedures for the determination of trace amounts of cadmium. Cadmium is a familiar hazardous pollutant in the ecological system. It is an element that is relatively sparse in the earth’s crust, but poses a serious threat to human and animal health. As a result of human activities, cadmium has become the main chemical pollutant of the environment, and as it is used in many technology processes in various industries and agriculture, its presence is found in air, water, and soil as well as in plants and animal tissues. In industry, cadmium is used for the production of dyes and plastic stabilizers, artificial and galvanic protective coatings, solders and alloys, and cadmium bars. It is also used for the production of alkaline nickel-cadmium batteries, fireworks, and fluorescent paints [20]. Fertilizers (e.g., superphosphates) that are contaminated with this metal in an amount from 10 to 100 mg/kg are a significant source of cadmium in the environment. Its long-term and widespread use leads to continual cadmium contamination of the soil [21]. Once introduced into the environment, cadmium is not subject to degradation and remains in constant circulation. Its long half-life translates into the accumulation of this element in the organisms of plants, animals, and humans. Environmental exposure factors can lead to the absorption of large amounts of cadmium and the toxic effects of this element on the body. In living organisms, even in small amounts, it causes liver diseases, kidney and cardiovascular dysfunction, toxic effects in Alzheimer’s disease, and carcinogenic effects on humans [22]. Therefore, it is crucial to obtain information on the amount of Cd(II) ions in real environmental samples as their toxicological effect depends on their concentration and the form of the compound in which cadmium occurs [23].

In our research, we focused on the determination of Cd(II) in water environmental samples, and we wanted to use working electrodes of a new generation for this purpose, allowing for excellent signal reproducibility and high sensitivity of determinations. In the research, anodic stripping voltammetry (ASV) was used, which allows the above-mentioned advantages of film modified solid electrodes to be exploited. Stripping voltammetric analysis methods are widely used in trace analysis of various metals and successfully used to monitor environmental samples [12,13,24,25,26,27,28,29]. Additionally, these techniques have often been used to designate cadmium as heavy metal. Abbasi et al. [30] summarized the literature on cadmium determination using the striping voltammetry technique up to 2011. In their work, Rojas-Romo et al. [31] summarized the electroanalytical methods applied for Pb(II) and Cd(II) determination using different types of working electrodes and anodic stripping voltammetry. The vast majority of these papers describe the determination of cadmium ions simultaneously with other elements, most often lead. Here, we determine cadmium without accompanying ions. Table 1 compares the proposed procedure with the publications concerning the determination of Cd(II) ions in the works from recent years using the ASV technique.

As we can see, our procedure has the lowest detection limit compared to other ASV procedures for the determination of Cd(II) ions published in recent years. We achieved this due to the use of new generation copper modified electrodes, CuF/CN/SPE and CuF/GCE, in cadmium analysis for the first time. We obtained detection limits even lower than with the use of mercury electrodes, which, as is well known, enable determination of one of the lowest detection limits in voltammetric methods. The elimination of mercury electrodes from research is another aspect that supports the development of other electrochemical sensors using non-toxic metals. It is, therefore, a major advantage of the tested method described here.

## 2. Materials and Methods

### 2.1. Apparatus

A μAutolab analyzer (EcoChemie, Utrecht, The Netherlands) with GPES software was used to perform voltammetric studies. The three-electrode system used for measurement consisted of a glassy carbon working electrode and a modified carbon nanotubes screen-printed working electrode (GCE, 1 mm diameter, and CN/SPE, 4 mm diameter), an Ag/AgCl (saturated NaCl) reference electrode (AutoLab), and platinum wire as an auxiliary electrode (AutoLab). The surfaces of the working electrodes were modified before each measurement in situ with copper. The studies were conducted in a volumetric cell (10 mL volume). The glassy carbon electrode (AutoLab) was polished daily on 2000 grit sandpaper, and afterwards it was polished using 0.3 μm alumina slurry on a Buehler polishing pad and immersed for 30 s in an ultrasonic bath. The modified carbon nanotubes screen-printed electrode was used without any special preparation in the form in which it was purchased (nLab). FEI Quanta 3D FEG scanning electron microscope (SEM) equipped with an energy dispersive X-ray spectrometer EDX Octane Elect Plus was used to accurately identify surface morphology and to take images of the electrode surfaces.

### 2.2. Reagents

The supporting electrolyte was prepared by diluting concentrated hydrochloric acid to 0.1 M HCl (Suprapure Merck). Standard cadmium of 1 g/L was purchased from Fluka (Buchs, Switzerland). The working solution of Cd(II) with a lower concentration of 1×10^−4^ M was prepared from standard cadmium in 0.01 M HNO_3_ solution. The interference effect was tested using standard stock solutions of 1 g/L of Al(III), As(III), As(V), Ca(II), Cr(III), Cr(VI), Fe(III), Mg(II), Mn(II), Ni(II), Pb(II), W(VI), Zn(II), Ti(IV), Sb(III), Mo(VI), Sn(IV), Se(IV), In(III), and Ga(III) from Fluka. The solution of Triton X-100 (nonionic surfactant), SDS (anionic surfactant), and CTAB (cationic surfactant) were purchased from Fluka, whereas HF (humic acids) was obtained from Aldrich. FA (fulvic acids) and NOM (natural organic matter) from the Suwannee River were purchased from the International Humic Substances Society. Rhamnolipids (biosurfactant) and Amberlite XAD-7 resin were obtained from Sigma. The resin was prepared by rinsing it four times in distilled water and drying at 50 °C before use. All solutions were made using ultra-purified water supplied by a Milli-Q system (Millipore, London, UK).

In the research, certified reference materials were used such as: TM-25.5 (environmental matrix reference material, Environment and Climate Change, Ottawa, ON, Canada), SPS-SW1 (surface water, Spectrapure Standards As, Oslo, Norway), and SPA-WW1 (waste water, Spectrapure Standards As, Oslo, Norway). 

### 2.3. ASV Procedure of Cadmium Determination

For both used electrodes, CuF/CN/SPE and CuF/GCE, the measurements were performed under optimum conditions using hydrochloric acid at a concentration of 0.1 M containing 2 × 10^−4^ M Cu(II). The experiments were performed using differential pulse anodic stripping voltammetry (DP-ASV) in the following sequence of potentials: +0.4 V for 10 s and −0.7 V for 60 s for CuF/GCE, and +0.4 V for 10 s and −0.75 V for 60 s for CuF/CN/SPE. The first step was performed to electrochemically clean the working electrode. The potential and time of electrochemical cleaning had been optimized and successfully applied in the previous work using CuF/GCE [1,4], and in this work it also proved to be effective in removing traces of earlier measurements from the surface of the solid electrode. During the second potential (accumulation potential), in situ plated copper on the surface glassy carbon electrode and cadmium on the surface of the produced copper film were deposited simultaneously. After a deposition time of 60 s, the differential pulse stripping voltammogram was recorded, after 5 s equilibration time, while the potential was scanned from −0.7 V to −0.4 V for CuF/GCE and from −0.8 V to −0.5 V for CuF/CN/SPE, with a pulse time of 10 ms and a pulse height of 50 mV. The measurements were conducted on the non-deareated solution with no apparent effect on the cadmium signal. During all steps, the solution was stirred using a magnetic stirring bar. The intensity of the obtained signal was proportional to the concentration of Cd(II) in the sample solution.

### 2.4. Procedure of Preliminary Mixing with Resin

When conducting studies on real water samples, one should take into account the possibility of a negative impact on the measurements of organic substances and surfactants that may be present in such samples. The organic substances and surfactants can adsorb on the electrode surface, subsequently blocking electroactive sites. In our previous studies [14,24], we have proved that such interferences can be effectively eliminated using Amberlite XAD-7 resin with adsorption properties. During the procedure of preliminary mixing with resin, the interfering substances are adsorbed onto the resin, and consequently the CuF/CN/SPE and CuF/GCE electrodes are not blocked and the Cd(II) ions can be efficiently adsorbed on the modified electrode surface. Due to this, the determination can be carried out directly from a natural sample without negative organic matter interferences. An additional advantage is the fact that, in ASV procedures, the resin can be added directly to the measuring cell. In the case of adsorptive stripping voltammetry procedures (AdSV), mixing with the resin has to be performed in an additional step before the actual measurement [14,24]. This is due the fact that, in the case of AdSV methods, it is necessary to introduce a complexing agent into the vessel and, as it has been proven, the determined metals in the form of complexes are often adsorbed on the resin, which results in lower results. In the case of the ASV method, it is not necessary to introduce a complexing agent and the determined metal is not adsorbed on the resin. In this case, 0.1 g of resin was added directly to the measuring cell and the determinations were performed as described in Section 2.3.

## 3. Results and Discussion

In the earlier literature [1,2,3,4], it was documented that the copper film electrode can be another interesting alternative to mercury electrodes, apart from the lead film electrode [13,14] and the bismuth film electrode [31,41]. As proven in this work, a copper film can be generated on both the GCE and CN/SPE substrate. It enables the analysis to be transferred to field conditions, which provides quick and cheap direct analysis of environmental samples. In order to achieve the best performance and lowest detection limit, an optimization study was performed. The parameters influencing the height of the obtained signal were optimized: the pH and concentration of the supporting electrolyte, the concentration of copper, the deposition potential and time, and the pulse time and pulse height of the stripping voltammetry measurement of the trace concentration of Cd(II) ions. The optimization process was carried out first for the electrode CuF/GCE.

### 3.1. Effect of Compositionand Concentration of Supporting Electrolyte

The type and pH of the basic electrolyte used in anodic stripping voltammetry measurements is of great importance for the sensitivity, stability, and repeatability of analytical signals. Several solutions that can act as the supporting electrolyte were tested, including ammonia buffer, acetate buffer, phosphorus buffer, hydrochloric acid, perchloric acid, and acetic acid. In the previous study that used CuF/GCE as a working electrode, 0.1 M HCl with 0.4 M NaCl [1,4] or 0.01 M HCl [2] was selected as a supporting electrolyte. Additionally, in the case of this work, after preliminary tests and attempts to obtain a signal, hydrochloric acid was selected from among the above-mentioned reagents. In all cases, the measurements were performed for a solution with a standard composition, a fixed concentration of 5 × 10^−8^ M Cd(II), 2 × 10^−4^ M Cu(II), and 0.1 M of the tested supporting electrolyte, and with a variable pH range in the case of the buffer solution. It was observed that only in the case of hydrochloric acid the cadmium signal was obtained, so this acid was used in further studies. 

In addition to the selection of the electrolyte, its concentration in the tested sample also had to be adjusted. The concentration of hydrochloric acid was examined in the range from 0.05 to 0.4 M. The studied solution contained, as previously, 5 × 10^−8^ M Cd(II), 2 × 10^−4^ M Cu(II), and an appropriate amount of HCl. It was noted that the highest, narrowest, and symmetric peak was obtained at a concentration of 0.1 M hydrochloric acid in the solution. At a lower concentration of HCl in the solution, the cadmium peak was lower, while at a higher concentration of HCl in the solution, the peak initially remained the same and then decreased. In the next measurement, the hydrochloric acid concentration of 0.1 M was selected. 

### 3.2. Effect of Copper Concentration

**Figure 1:**
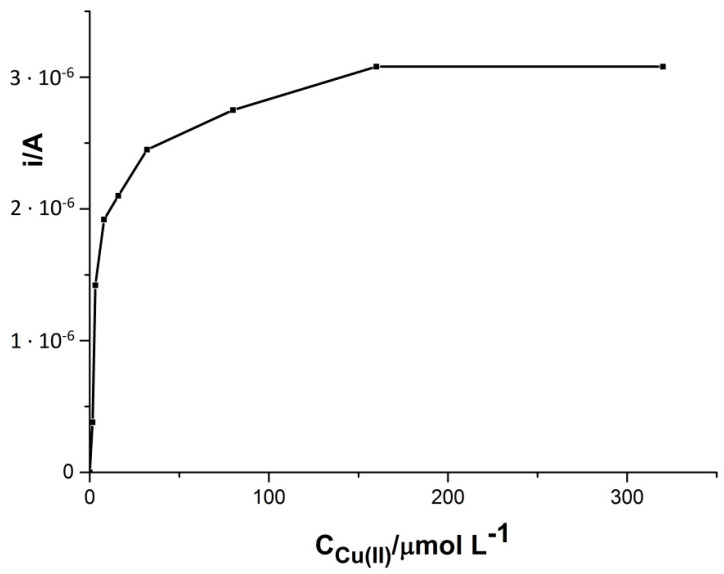
Influence of copper concentration on the Cd(II) signal. Concentration of Cd(II) 5 × 10^−8^ M. Accumulation potential −0.7 V and accumulation time 60 s.

The influence of the concentration of Cu(II) in the measured solution used to create the thin film on the surface of the solid electrode on the cadmium signal is shown in Figure 1. As shown, copper concentration affects the signal obtained by voltammetric technique. The analysis was carried out with the solution containing a fixed concentration of 5 × 10^−8^ M Cd(II) and 0.1 M HCl with a variable concentration of Cu(II) from 1.6 × 10^−6^ to 3.2 × 10^−4^ M. The stripping of cadmium sharply increased in the concentration range between 8 × 10^−6^ and 4 × 10^−5^ M; at a higher concentration of Cu(II), the cadmium signal continued to increase, but slightly, to a concentration of 1.6 × 10^−4^ M, and then remained constant. Taking into account the above considerations, the optimal concentration of copper in the test objects was assumed to be 2 × 10^−4^ M. Additionally, using the Randles-Sevcik equation [42], the active surface areas of the working electrode surfaces were calculated. Using this Equation (1), the peak current (I_p_) is defined as:

(1) $$I_{p}=0.4463{\left(\frac{F^{3}}{\mathrm{RT}}\right)}^{1/2}{\mathrm{An}}^{3/2}D^{1/2}C_{o}\nu^{1/2}$$

where: F—Faraday constant (F = 96 485 C mol^−1^), T—the absolute temperature (T = 298 K), R—the universal gas constant (R = 8.314 J mol^−1^ K^−1^), A—the electrode surface area (cm^2^), *n*—the number of electrons involved in the redox reaction (*n* = 2), D—diffusion coefficient (D = 7.2 × 10^−6^ cm^2^ s^−1^), and C_o_—the concentration of Cu(II) (2 × 10^−4^ M). For the CuF/GCE working electrode geometric area of the surface was equal to 0.00785 cm^2^, while the active surface area of the glassy carbon electrode modified with copper equals to 0.00017 ± 0.00001 cm^2^, number of repeated measurements = 3 (*n*). The smaller active area than the geometric area of the electrode confirms the fact that the active sites on the electrode surface are copper sites. The area between the accumulated copper remains inactive.

### 3.3. Conditions of Accumulation Potential and Time

In order to check the effect of the accumulation potential on the measurements, tests were carried out with the solution containing 5 × 10^−8^ M Cd(II), 2 × 10^−4^ M Cu(II), and 0.1 M HCl. During the accumulation potential stage, a copper film is formed and, at the same time, cadmium is accumulated in the form of Cd(0) as a result of the reduction in its Cd(II) ions. In the optimization, the accumulation potential was changed over the range of −0.9 to −0.5 V. The obtained results showed that the cadmium signal was visible for the accumulation potential range from −0.8 to −0.65 V, and the highest peak was obtained at the accumulation potential of −0.7 V. Therefore, for further experiments, the accumulation potential equal to −0.7 V was selected as the most appropriate potential for anodic stripping voltammetry determination of Cd(II) ions.

**Figure 2:**
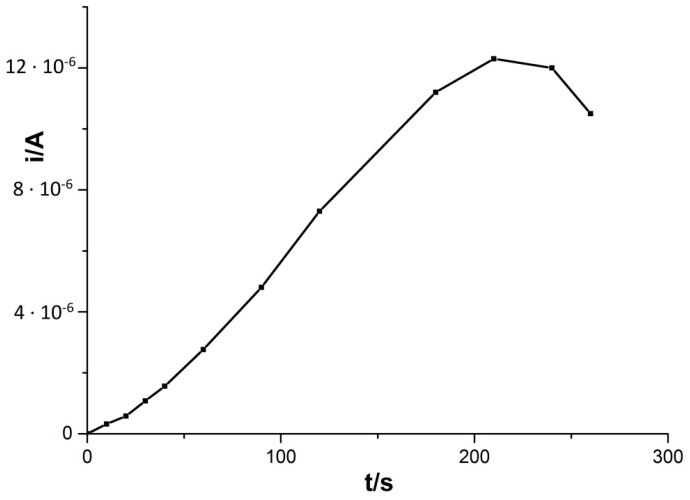
Influence of accumulation time on the Cd(II) signal. Composition of solution 5 × 10^−8^ M Cd(II), 2 × 10^−4^ M Cu(II), and 0.1 M HCl. Accumulation potential −0.7 V.

After adjusting the accumulation potential, the accumulation time was optimized. This parameter has a pronounced effect on sensitivity in stripping techniques. This influence was measured in the accumulation time range 0–260 s. In the tested solution, the concentration was 5 × 10^−8^ M Cd(II), 2 × 10^−4^ M Cu(II), and 0.1 M HCl. The influence of accumulation time on the Cd(II) peak current is presented in Figure 2. The accumulation potential was −0.7 V. The value of the voltammetric signal increased almost linearly with the accumulation time prolonged to 210 s. For the longer accumulation time, we can observe a reduction in the cadmium peak and the blurring of its shape. Thus, an accumulation time of 210 s was used as optimal in constructing the calibration curve and calculating the limit of detection, RSD, and the correlation coefficient. However, to shorten the measuring time, an accumulation time of 60 s was used in the measurements during the optimization procedure, interfering testing, and tests with certified reference materials. 

### 3.4. Pulse Time and Pulse Height

**Figure 3:**
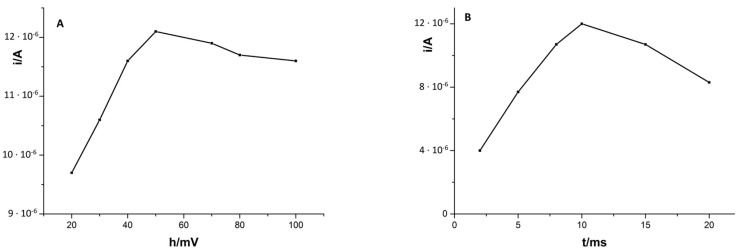
Influence of pulse height (**A**) and pulse time (**B**) on the Cd(II) signal. Composition of solution 5 × 10^−8^ M Cd(II), 2 × 10^−4^ M Cu(II), and 0.1 M HCl. Accumulation potential −0.7 V, accumulation time 210 s.

The pulse time and pulse height also have effects on the cadmium peak intensity, so they were also examined. The pulse time was examined from 2 to 20 ms, and it turned out that, with an increase in pulse time above 10 ms, the signal of Cd(II) decreased, and hence for further tests the value of 10 ms was chosen. The variation of the pulse height between 20 and 100 mV showed that with the increase in pulse height to 50 mV, the peak current of cadmium increased linearly. In the higher values, the signal of Cd(II) undergoes gradual blurring. Figure 3 shows the obtained results of cadmium peak current on pulse height (A) and pulse time (B).

### 3.5. Analytical Characterization

**Figure 4:**
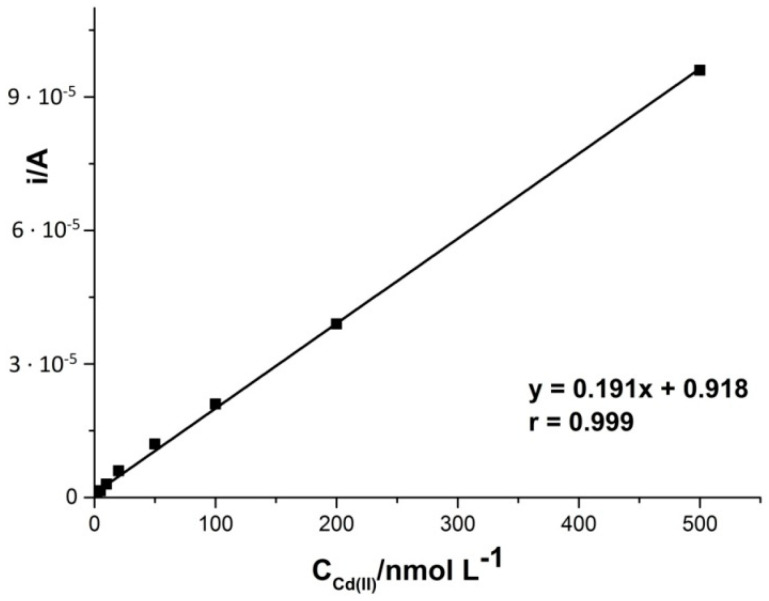
Linear range of the cadmium calibration curve. DPV parameters: accumulation time 210 s, accumulation potential −0.7 V.

**Figure 5:**
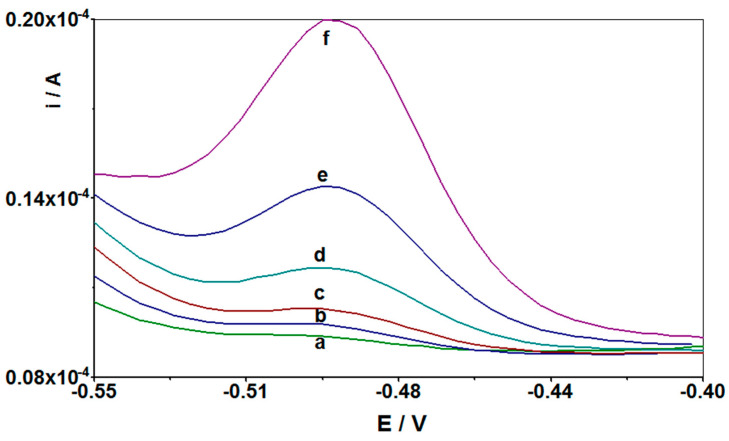
Differential pulse voltammograms obtained in the course of Cd(II) determination at the GCE working electrode recorded for solutions containing: (**a**) background: 1 mL 1 M HCL + 125 µL 1 g/L Cu(II) and distilled water; (**b**) as (**a**) + 1 × 10^−9^ Cd(II); (**c**) as (**a**) +2.5 × 10^−9^ Cd(II); (**d**) as (**a**) + 5 × 10^−9^ Cd(II); (**e**) as (**a**) + 1 × 10^−8^ Cd(II); and (**f**) as (**a**) + 2.5 × 10^−8^ Cd(II). Accumulation potential −0.7 V and accumulation time 210 s.

Based on the previously optimized parameters, such as concentration and type of the supporting electrolyte, copper concentration, accumulation potential and time, and pulse time and height, a series of measurements was carried out to prepare a calibration curve. For this purpose, the solution was prepared: 0.1 M HCl, 2 × 10^−4^ M Cu(II), to which cadmium additives were added during the measurements with an accumulation time of 210 s and with an accumulation potential of −0.7 V. It was found that the intensity of the peak current derived from cadmium ions increased linearly (correlation coefficient r = 0.999) in the concentration range from 5 × 10^−10^ to 5 × 10^−7^ M. The limit of detection calculated from the calibration curve is equal to 1.7 × 10^−10^ M, with the equation y = 0.191x + 0.918, where y is the peak current (μA) and x is Cd(II) concentration (nM). The sensitivity calculated for comparison with other papers [43] was 1123.529 μA nM^−1^ cm^−2^. The relative standard deviation (RSD) for all measured concentrations of cadmium from the linear range of the calibration graph was 4.2% (*n* = 5). Figure 4 presents the linear range of the Cd(II) calibration curve. Figure 5 shows selected voltammograms obtained when creating a calibration curve for low concentrations of cadmium in the sample.

The reproducibility of the peak current was also determined by successive measurements (*n* = 5) of the signal of 5 × 10^−9^ M Cd(II) and was assessed from the experiments performed in five consecutive days as RSD, which was 3.2%.

### 3.6. Interferences

Before attempting an analysis of real water samples, the influence of potential interference substances and ions on the analytical signal of 5 × 10^−8^ M Cd(II) was investigated. Two major sources of interference were examined: other metal or metalloid ions and organic substances, surfactants. Interference from other metal or metalloid ions could cause the blocking of the working electrode surface or create intermetallic compounds with other components of the tested solution causing a reduction or complete disappearance of the cadmium signal. The effects of the influence of co-existing metal or metalloid ions were examined using a fixed concentration of Cd(II) with different amounts of foreign ions under standard optimized conditions. The result showed that an up to 200-fold excess of Al(III), As(III), As(V), Ca(II), Cr(III), Cr(VI), Fe(III), Mg(II), Mn(II), Ni(II), W(VI), Zn(II), Ti(IV), Sb(III), Mo(VI), Sn(IV), Se(IV), In(III), and Ga(III) did not have any significant effect on the Cd(II) peak current. The addition of a 100-fold excess of Pb(II) and Sn(IV) caused a 50 ± 3% decrease in the cadmium signal. 

Surfactants and humic substances are other types of interfering substances occurring in natural water samples. They can adsorb on the surface of the electrode, which reduces access to it and may make it difficult to form a metallic film on it [44]. In order to investigate the effect of these substances on the cadmium peak current, experiments with non-ionic surfactant Triton X-100, cationic surfactant CTAB (cetyltrimethylammonium bromide), anionic surfactant SDS (sodium dodecyl sulfate), and biosurfactant Rhamnolipids were carried out. As humic substances, humic acid (HA), fulvic acid (FA), and natural organic matter (NOM) were used in the measurements. In the case of determination of Cd(II) ions, only three of the above-mentioned substances caused a decrease and, consequently, at higher concentrations, the disappearance of the cadmium peak. As observed already, a concentration of 2 ppm CTAB caused a reduction in the cadmium peak by about 80%, while the addition of 2 ppm HA and FA decreased the signal by about 60%. In the case of other organic substances, additions up to 30 ppm (NOM) and 50 ppm (Triton X-100, SDS, Rhamnolipid) did not significantly affect the cadmium signals, only a deterioration of the peak shape was observed with large amounts of the additives. Taking into account the above considerations and the previously presented lack of negative influence on the measurements of foreign metal ions, the lack of interference from NOM, Triton X-100, SDS, and Rahmnolipid potentially present in natural samples is a great advantage of the described procedure. This makes it possible to use the Cd(II) determination procedure on the CuF/GCE electrode in direct tests from natural samples without the need to prepare them for analysis, and to use the CuF/CN/SPE electrode to conduct research in field conditions. This significantly reduces the costs and time of the performed determinations.

**Figure 6:**
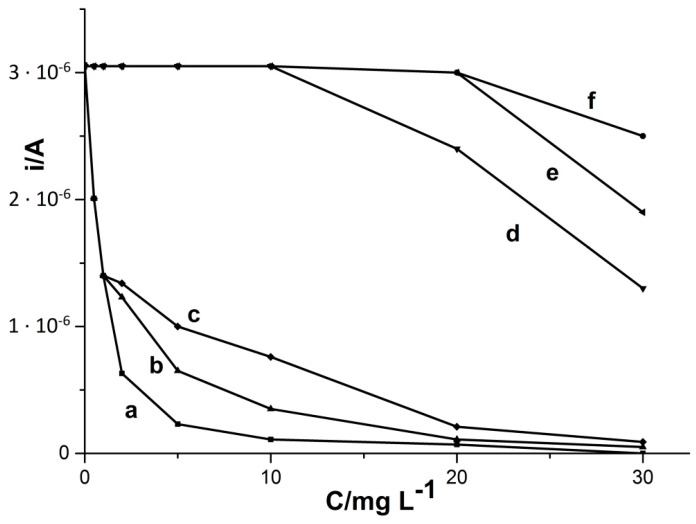
Influence of CTAB (**a**,**f**), HA (**b**,**d**), and FA (**c**,**e**) on the cadmium peak intensity using the procedure without (**a**–**c**) and with (**d**–**f**) preliminary mixing with Amberlite XAD-7 resin. Concentration of Cd(II) 5 × 10^−8^ M, accumulation potential −0.7 V, and accumulation time 60 s.

In case of CTAB, FA, and HA, in order to eliminate the negative influence on the signals, the procedure of preliminary mixing the test sample with the resin Amberlite XAD-7, having adsorption properties, was used. This developed method is described in the literature on the subject [14,24,36]. All steps of preliminary mixing with the resin used in this work are described earlier in Section 2.4. Figure 6 presents the results obtained before and after application of preliminary mixing with the resin for the interfering substances CTAB, HA, and FA. In Table 2, we can also see the results obtained when using preliminary mixing with the resin Amberlite XAD-7 and, for comparison, without using this procedure. Thanks to this method, an undisturbed cadmium signal was obtained even at 20 ppm CTAB and FA, and at 10 ppm HA in the tested sample. Thus, we can see a significant improvement and the effectiveness of the resin used in removing interference.

### 3.7. Impact of Temperature

In the next stage of the research, it was checked whether the increase in temperature from 20 to 60 °C had an impact on cadmium signals obtained using new modifications of the CuF/CN/SPE and CuF/GCE electrodes. For this purpose, a series of measurements were carried out for the solution containing a constant cadmium concentration of 5 × 10^−8^ M, 0.1 M HCl, and 2 × 10^−4^ M Cu(II) at 20, 30, 40, 50, and 60 °C. For this purpose, an appropriately designed 10 mL voltammetric cell connected to a thermostat was used, which allowed the desired temperature to be maintained. For each temperature, a series of 5 measurements was carried out to check the stability of the obtained signal. The obtained results showed that temperature did not affect the cadmium signal, which means that it did not affect the process of creating new types of electrodes modified with copper: CuF/CN/SPE and CuF/GCE. This is a great advantage of these electrodes that can work in a wide temperature range without adversely affecting the process of surface modification of the working electrodes.

In subsequent studies, it was investigated whether the increase in temperature may improve the elimination of interference from CTAB, HA, and FA, and increase the permissible concentrations of other organic substances, so that, even at higher concentrations in the samples, they would not affect the cadmium peak current. It was also investigated whether the increase in temperature may affect the better performance of the Amberlite XAD-7 resin in the process of removing interferences from organic substances. The measurements were carried out using the conditions and composition of the solution as before: 0.1 M HCl, 2 × 10^−4^ M Cu(II), 5 × 10^−8^ M Cd(II), potential −0.7 V, time 60 s, and an appropriate quantity of organic substances and resin. The temperature was varied from 20 to 60 °C during the measurements, performing five repetitions at a given temperature. Based on the obtained results, it was proven that a temperature rise to 50 °C reduces the negative impact of organic substances on the cadmium peak while, when the resin was used, greater recoveries were obtained than at 20 °C. At higher temperatures (60 °C), the cadmium signal slightly decreased. The results for the influence of CTAB, HA, and FA at various temperatures on the voltamperometric cadmium peak current are collected in Table 3. As can be concluded from the obtained data, the use of elevated temperature (up to 50 °C) allows for better sensitivity of the determinations in the presence of interfering substances. The improvement of the signal is not significant but, with a high presence of organic substances in the samples, it is possible to additionally reduce these interferences by manipulating the temperature.

### 3.8. Procedure with CuF/CN/SPE Electrode

#### 3.8.1. Morphological, Structural, and Compositional Information of the Electrode Materials

**Figure 7:**
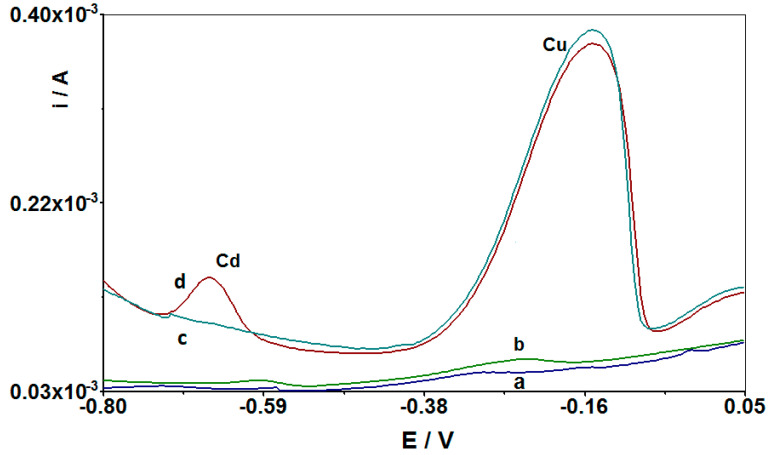
Comparison of differential pulse voltammograms obtained in the course of Cd(II) determination at the CuF/CN/SPE working electrode: (**a**) background, without addition of Cu(II) and Cd(II); (**b**) as (**a**) + 1 × 10^−7^ M Cd(II); (**c**) as (**a**) + 2 × 10^−4^ M Cu(II); and (**d**) as (**b**) + 2 × 10^−4^ M Cu(II). Accumulation potential and time was −0.7 V and 210 s, respectively.

After the optimization of the procedure of cadmium determination using CuF/GCE as a working electrode, additional studies were performed using novel modified screen-printed electrodes CuF/CN/SPE. It turned out that the developed test method can also be effectively applied by using the CuF/CN/SPE electrode without a significant change in the measurement parameters. A novel copper film with carbon nanotubes modified screen-printed electrode was used in the tests. The SPE electrodes are now very popular and are often used in voltamperometric determinations [5,6,7,8,9,10,15]. They are valued primarily for their reproducibility and sensitiveness, effectiveness in analysis, a large active surface, excellent electrical conductivity, and good chemical stability. Additionally, they combine the three-electrode system into one system, which reduces the costs of analysis, and due to their small size they enable analyses to be carried out in the field. Figure 7 presents the voltammograms obtained for the CuF/CN/SPE electrode with the appropriate additives: with cadmium without copper, after adding copper to the solution, and in a solution with only copper without cadmium. As can be seen, without copper, there is no signal from the cadmium present in the solution. Only after adding copper to the tested solution two peaks appear in the voltammogram, one from Cd(II) and one from Cu(II) ions. This is confirmed by the fact that a copper film is formed on the surface of the CuF/CN/SPE electrode, which allows the accumulation of cadmium ions on its surface.

**Figure 8:**
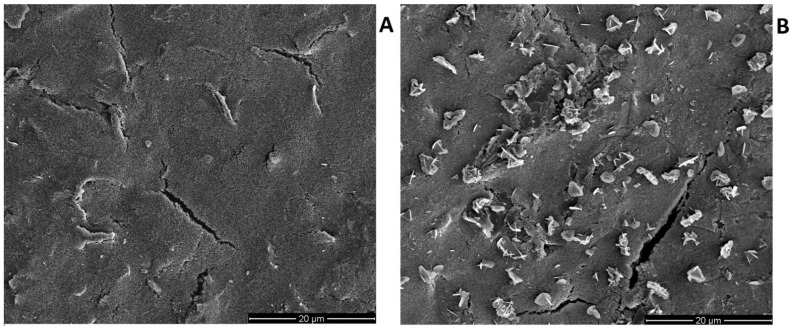
Images obtained through scanning electron microscope for the bare (**A**) and modified with copper film (**B**) CN/SPE working electrode. Copper film modified electrode was prepared in situ from the water solution containing 0.1 M HCl and 2 × 10^−4^ M Cu(II).

**Figure 9:**
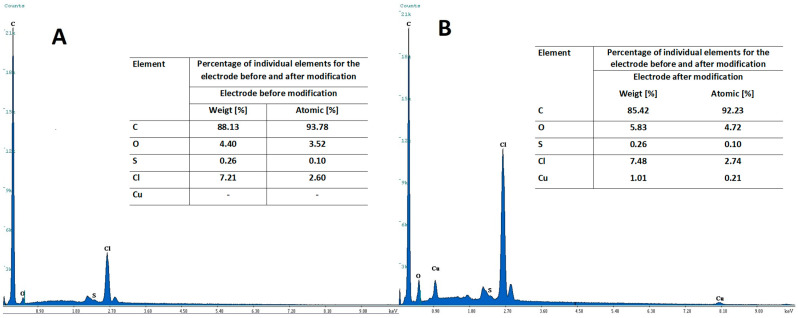
EDX spectra obtained for electrode non modified (**A**) and modified with copper (**B**) from the solution containing 0.1 M HCl and 2 × 10^−4^ M Cu(II).

Additionally, Figure 8 presents images of the morphology of CuF/CN/SPE electrode surface unmodified (A) and after copper film modification (B). The images obtained by scanning electron microscope display the effect of covering the working electrode surface with copper, and it was confirmed after comparison of the images of bare and in situ modified electrode surface. After the in situ deposition of copper bright points (clusters of copper) appeared on the electrode surface (Figure 8B). This was confirmed by EDX analysis, which revealed the presence of certain amounts of Cu on the modified electrode surface, and no Cu on the bare electrode surface. The results of the EDX analysis are shown in Figure 9.

As described earlier for CuF/GCE electrode in Section 3.2 using the Randles-Sevcik Equation (1), active surface areas of the CuF/CN/SPE electrode surfaces were calculated [42]. For the CuF/CN/SPE, geometric area of the surface was equal to 0.12560 cm^2^, while the active surface area of the carbon nanotubes screen-printed electrode modified with copper equals to 0.04673 ± 0.00170 cm^2^ (*n* = 3). The smaller active area than the geometric area of the electrode confirms the results obtained from morphology images and EDX analysis. The active sites of the electrode surface in this case are the copper sites, and the sites outside the copper are in active for cadmium accumulation. This is consistent with the voltammograms presented in Figure 7 confirming that, without copper on the electrode, cadmium does not undergo accumulation.

#### 3.8.2. Analytical Parameters

**Figure 10:**
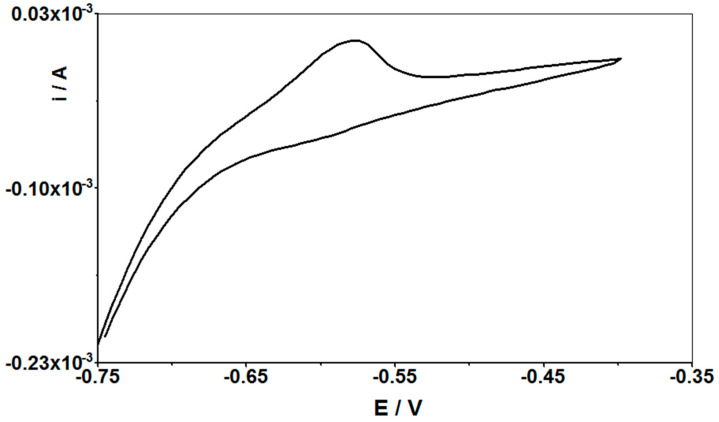
Cyclic voltammograms of 5 × 10^−8^ M Cd(II) in 0.1 M HCl and 2 × 10^−4^ M Cu(II) solution at the CuF/CN/SPE electrode, for scan rate of 1 V s^−1^.

The parameters influencing the Cd(II) signal height were optimized. The same parameters were tested as in the case of the CuF/GCE electrode: the pH and concentration of the supporting electrolyte, the concentration of copper, the deposition potential and time, and the pulse time and pulse height. The measurements were performed with a fixed concentration of Cd(II) 5 × 10^−8^ M. The selected composition of the test solution was the same as before: 0.1 M HCl, 2 × 10^−4^ M Cu(II). After the tests, it was confirmed that the most optimal cadmium signal was obtained for the same parameters as for GCE, and only a slight change can be made to the potential for accumulation of cadmium ions on the surface of the modified CuF/CN/SPE electrode, changing it to −0.75 V. At this potential, a slight improvement in the shape and height of the peak was obtained, but the −0.7 V potential, which generates equally high signals and is equally reproducible, can also be used successfully. The accumulation time remained the same as before and it was 60 s. Cyclic voltammetry (CV) analysis was also performed, and it was proven that the cadmium accumulation process on the working electrode is irreversible. An example voltammogram is shown in Figure 10.

#### 3.8.3. Analytical Characterization

**Figure 11:**
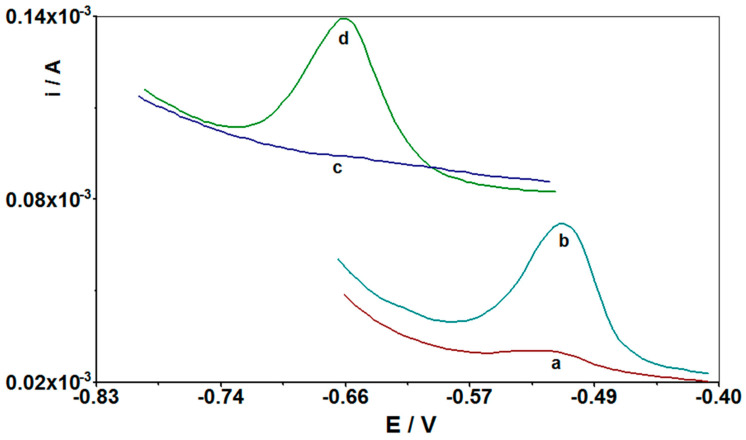
Comparison of differential pulse voltammograms obtained in the course of Cd(II) determination at the electrode CuF/GCE (**a**,**b**) and CuF/CN/SPE (**b**,**c**): (**a**) background for CuF/GCE; (**b**) as (**a**) + 1 × 10^−7^ M Cd(II); (**c**) background for CuF/CN/SPE; and (**d**) as (**c**) + 1 × 10^−7^ M Cd(II). Accumulation potential and time for CuF/GCE was −0.7 V and 210 s; accumulation potential and time for CuF/CN/SPE was −0.75 V and 210 s.

The detection limit obtained in the case of the CuF/CN/SPE electrode was slightly lower from that for the CuF/GCE electrode, amounting 1.3 × 10^−10^ M, while the linearity range of the calibration curve ranged from 3 × 10^−10^ to 3 × 10^−7^ M with an accumulation time of 210 s and accumulation potential of −0.75 V. The equation of the calibration curve was equal to y = 0.333x + 0.396, where y is the peak current (μA) and x is Cd(II) concentration (nM) with correlation coefficient r = 0.999. As for the CuF/GCE electrode, the sensitivity was calculated for the CuF/CN/SPE electrode and was 7.126 μA nM^−1^ cm^−2^. Figure 11 presents the comparison of the voltammograms obtained for the CuF/GCE and CuF/CN/SPE electrodes. The conducted research shows that the CuF/GCE or CuF/CN/SPE novel modified electrodes can be applied interchangeably for the determination of cadmium using the voltammetric procedure developed in this study without loss in sensitivity or reproducibility of signals. Both electrodes give similar effects, but CuF/CN/SPE has a lower limit of detection and can be successfully used in field studies of real samples, which is an extremely important aspect in environmental analysis and a great advantage of the described research work. In addition, the CuF/CN/SPE electrode is readily commercially available, making it affordable for any scientist. 

### 3.9. Analytical Application

In order to validate the developed procedure, tests were carried out with certified reference materials. The certified references materials TM-25.5 (environmental matrix), SPS-WW1 (waste water), and SPS-SW1 (surface water) were selected. The advantage of these materials is that they contain between 13 and 45 different trace elements, including cadmium. The cadmium concentration in these materials is 24 ng mL^−1^ (TM-25.5), 20 ng mL^−1^ (SPS-WW1), and 0.52 ng mL^−1^ (SPS-SW1). The concentration of the remaining components of the solutions ranged from 0.5 ng mL^−1^ to 2000 ng mL^−1^; these matrices reflect the composition of environmental samples very well. The measurements were performed using the standard addition method. In the case of SPS-WW1 and SPS-SW1, and an appropriate amount of NaOH was additionally added to neutralize the solution as these materials contain nitric acid. All experiments were performed in five replicates. The recoveries were between 92.25% and 107.69%, whereas the relative standard deviations between 5.8% and 6.5%, which indicates good accuracy of the proposed method. Table 4 presents the results of Cd(II) determination in the certified reference materials.

To confirm the applicability of this procedure to the analysis of environmental samples, the proposed method was applied in the determination of Cd(II) in natural water samples collected from eastern areas of Poland. Tap water and rainwater were also tested. The voltammograms recorded for those samples did not exhibit any cadmium signal, which proves that the concentration of cadmium in the tested samples was below the limit of detection. To confirm the possibility of determining Cd(II) ions in such samples, the analyzed samples were spiked with cadmium. The standard addition method was used to calculate the recovery value. All experiments were carried out in five replicates. The recoveries were between 96.54% and 101.50%, whereas the relative standard deviations between 3.5% and 4.3%, which indicates good accuracy of the developed method. Table 5 presents the results of Cd(II) determination in natural water samples. 

## 4. Conclusions

In this work, the authors present the applicability of the copper film modified glassy carbon electrode and the novel copper film with carbon nanotubes modified screen-printed electrode for anodic stripping voltammetric determination of trace concentrations of cadmium. This is the first work of this type devoted to the determination of cadmium ions on copper modified working electrodes. It was also the first time that the screen-printed electrode was successfully modified with copper. As it turned out, this approach allowed for significant reduction in the detection limits of ultratrace concentrations of cadmium ions. The CuF/GCE electrode was electrochemically deposited onto the glassy carbon solid electrode with simultaneous accumulation of Cd(II) ions. The CuF/CN/SPE electrode combines a solid electrode modified with carbon nanotubes, a platinum auxiliary electrode, and a silver reference electrode in the microcircuit. The advantage of these microelectrodes is better sensitivity and the possibility of using them in field experiments. They are also readily available in many variants depending on the needs of the researcher. Screen-printed electrodes have become an attractive analytical tool also due to the low production costs, appropriate levels of repeatability and their electrochemical properties. The use of the in situ created film electrode reduced the need for medium exchange after deposition of the metal, which significantly shortens the measurement time and reduces the consumption of chemical reagents. This method is simple, cheap, sensitive, selective, and fast, and does not require complicated apparatus. A very low detection limit for cadmium was obtained compared with other voltammetric techniques for the determination of Cd(II) ions (Table 1). In the literature we did not find any papers on the determination of cadmium on the CuF/GCE and with carbon nanotubes modified copper CuF/CN/SPE electrodes. The influence of various interfering substances, such as foreign metal ions and organic substances, was tested to check whether it would be possible to perform tests in aqueous environmental samples. Very satisfactory results were obtained, and in some cases the method using Amberlite XAD-7 resin with adsorptive properties was employed, which significantly improved cadmium recovery from the samples containing surfactants and humic substances. The influence of temperature on the performance of the modified electrodes was investigated, and it was proven that the cadmium signal is stable and the electrodes are perfectly reproducible over a wide temperature range. The method appears to be promising for its adoption in environmental research and field analysis.

## Figures and Tables

**Table 1:** Comparison of the proposed procedure with the previously reported voltammetric methods using ASV for the determination of Cd(II). The works are ranked according to the decreasing limit of detection.

Electrode	Accumulation Time	LOD	Sample	References
polyPCA/GE	125 s	0.142 μM	freshwater and real water	[32]
SWCNTs/Biomass/GCE	120 s	0.103 μM	real water	[33]
GQDs/NF/GCE	150 s	0.126 μM	bivalve mollusks	[34]
BOC/GCE	500 s	0.035 μM	tap water	[35]
Hg(Ag)FE	30 s	0.013 μM	real water	[36]
MFE/GCE	240 s	0.006 μM	the constituent parts of the illegal cigarettes	[37]
IL/GO/GCE	300 s	0.003 μM	tap water	[38]
MWCNT/GCE	not specified	0.002 μM	real water	[39]
BiFE/GCE	60 s	0.008 × 10^−1^ μM	real water	[31]
GO@Fe3O4@2-CBT/GCE	180 s	0.027 × 10^−2^ μM	real water	[40]
CuF/GCE	210 s	0.017 × 10^−2^ μM	real water	[this work]
CuF/CN/SPE	210 s	0.013 × 10^−2^ μM	real water	[this work]

polyPCA/GE—graphite electrodes modified with poly(*p*-coumaric acid), SWCNTs/Biomass/GCE—glassy carbon electrode modified by a mixture of single walled carbon nanotubes and biomass, GQDs/NF/GCE—glassy carbon electrode modified with graphene quantum dots and Nafion, BOC/GCE—glassy carbon electrode modified bismuth oxycarbide, Hg(Ag)FE—renewable mercury film silver-based electrode, MFE/GCE—glassy carbon electrode modified mercury film, IL/GO/GCE—glassy carbon electrode modified graphene oxide and ionic liquid, MWCNT/GCE—glassy carbon electrode multi-walled carbon nanotube electrode, BiFE/GCE—glassy carbon electrode modified bismuth film, and GO@Fe3O4@2-CBT/GCE—glassy carbon electrode modified with magnetic graphene oxide modified with benzothiazole-2-carboxaldehyde.

**Table 2:** Influence of CTAB, HA, and FA on the Cd(II) voltammetric signal using the procedure with and without preliminary mixing with Amberlite XAD-7 resin. Concentration of Cd(II) was 5 × 10^−8^ M.

Organic Substance	Maximum Allowable Concentration of Organic Substances That Does Not Interfere with the Cd(II) Signal (ppm)	
	Without Mixing with Resin	With Mixing with Resin
CTAB	1	20
HA	1	10
FA	1	20

**Table 3:** Influence of CTAB, HA, and FA on the Cd(II) voltammetric signal at different temperatures. Concentration of Cd(II) 5 × 10^−8^ M.

Organic Substance	Temperature (°C)	Maximum Allowable Concentration of Organic Substances That Does Not Interfere with the Cd(II) Signal (ppm)	
		Without Mixing with Resin	With Mixing with Resin
CTAB	20	1	20
	30	2	23
	40	3	28
	50	3	30
	60	2	24
HA	20	1	10
	30	1.5	14
	40	2.5	16
	50	2	20
	60	1	12
FA	20	1	20
	30	1.5	25
	40	2	28
	50	2	29
	60	1	25

**Table 4:** Analytical results of Cd(II) determination in the certified reference materials without and with addition of Cd(II) ions. The samples were examined using the standard addition method.

Sample	Cd(II) Content in Certified Reference Material (ng mL^−1^)	Cd(II) Found in Certified Reference Material (ng mL^−1^)	Recovery (%)	RSD (*n* = 5) (%)
TM-25.5	24	22.74	94.75	6.5
SPS-SW1	0.52	0.56	107.69	5.8
SPS-WW1	20	18.45	92.25	5.8

**Table 5:** Analytical results of Cd(II) determination in natural water samples. The samples were examined using the standard addition method.

Sample	Cd(II) Added (nM)	Cd(II) Found (nM)	Recovery (%)	RSD (*n* = 5) (%)
Tap water	50	49.23	98.46	3.7
Rain water	50	50.75	101.50	3.5
Bystrzyca river water	50	48.65	97.30	4.2
Lake Zemborzyce	50	48.27	96.54	3.8
San river water	50	50.09	100.18	4.3

## Data Availability

Data are available in a publicly accessible repository that does not issue DOIs; data are contained within this article. The data received in the project will be stored on a PC, and their backups will be stored on mobile devices (external drive). The official PC is connected to the KUL network, administered by DTI employees. Updates are performed daily. All data will be recorded in laboratory notes (during individual measurements, before entering them into a spreadsheet). The notebooks will be stored in the offices of those responsible for the implementation of the project. Raw data will be stored for a minimum of 10 years from the end of the project implementation.
